# Impact of Phrenic Nerve Repair Using Intercostal Nerve Graft on Diaphragm Function after Thoracic Tumour Resection

**DOI:** 10.1093/icvts/ivaf302

**Published:** 2025-12-14

**Authors:** Tetsuya Isaka, Yui Sueishi, Ikki Takada, Ryotaro Matsuyama, Chiaki Kanno, Takuya Nagashima, Kota Washimi, Seigo Katakura, Shuji Murakami, Haruhiro Saito, Hiroyuki Ito

**Affiliations:** Department of Thoracic Surgery, Kanagawa Cancer Center, Yokohama, Kanagawa 241-8515, Japan; Department of Thoracic Surgery, Kanagawa Cancer Center, Yokohama, Kanagawa 241-8515, Japan; Department of Thoracic Surgery, Kanagawa Cancer Center, Yokohama, Kanagawa 241-8515, Japan; Department of Thoracic Surgery, Kanagawa Cancer Center, Yokohama, Kanagawa 241-8515, Japan; Department of Thoracic Surgery, Kanagawa Cancer Center, Yokohama, Kanagawa 241-8515, Japan; Department of Thoracic Surgery, Kanagawa Cancer Center, Yokohama, Kanagawa 241-8515, Japan; Department of Pathology, Kanagawa Cancer Center, Yokohama, Kanagawa 241-8515, Japan; Department of Thoracic Oncology, Kanagawa Cancer Center, Yokohama, Kanagawa 241-8515, Japan; Department of Thoracic Oncology, Kanagawa Cancer Center, Yokohama, Kanagawa 241-8515, Japan; Department of Thoracic Oncology, Kanagawa Cancer Center, Yokohama, Kanagawa 241-8515, Japan; Department of Thoracic Surgery, Kanagawa Cancer Center, Yokohama, Kanagawa 241-8515, Japan

**Keywords:** phrenic nerve reconstruction, intercostal nerve grafting, diaphragm movement, respiratory function

## Abstract

**Objectives:**

This retrospective study investigated whether phrenic nerve repair with intercostal nerve graft affects postoperative diaphragmatic motion and respiratory function after thoracic tumour resection.

**Methods:**

We included 11 consecutive patients (reconstruction group: *n* = 8; nonreconstruction group: *n* = 3) who underwent thoracic tumour resection with phrenic nerve removal between October 2023 and March 2025. In the reconstruction group, the intercostal and phrenic nerves were connected end-to-end using 5-0 or 6-0 Prolene sutures. Postoperative respiratory function, inspiratory/expiratory diaphragm movement distance (IEDD), and inspiratory/expiratory lung area (IEA) ratio on chest X-ray were measured using SYNAPSE VINCENT and compared between the 2 groups.

**Results:**

No significant differences in age, sex, and side of phrenic nerve resected were observed between the 2 groups. IEDD ≥10 mm within 1 month postoperatively was seen in 4 (50%) patients in the reconstruction group. Mean IEDD on X-ray was 19.8 mm vs 4.1 mm (*P *= .013) at 1-3 months and 19.8 mm vs 4.4 mm (*P *= .031) at 4-6 months for the reconstruction and nonreconstruction groups, respectively. Mean IEA ratios were 1.16 vs 1.04 (*P *= .026) at 1-3 months and 1.19 vs 1.05 (*P *= .031) at 4-6 months, respectively. Postoperative respiratory function showed higher %VC (78% vs 56%, *P *= .008) and %FEV1 (72% vs 45%, *P *< .001) in the reconstruction group at 4-6 months.

**Conclusions:**

Phrenic nerve repair with intercostal nerve graft mitigated diaphragmatic dysfunction and maintained postoperative respiratory function after phrenic nerve resection.

**Clinical Registration Number:**

2024 Eki-102.

## Graphical abstract

**Figure:**
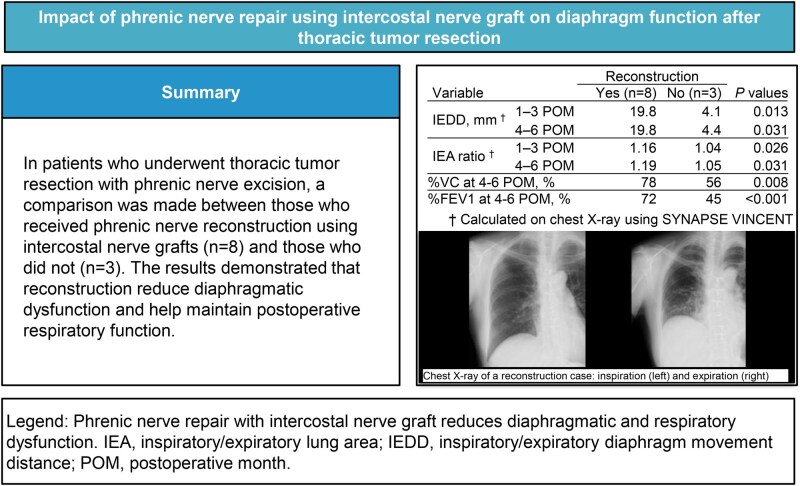

## INTRODUCTION

The diaphragm is the primary muscle responsible for respiration and is innervated by the phrenic nerve.^1^ During thoracic tumour resection, phrenic nerve palsy leading to diaphragmatic dysfunction occurs in approximately 2% of thymoma surgeries^2^ and 7% of lung cancer surgeries.^3^ In locally advanced thymoma, complete tumour resection often necessitates phrenic nerve sacrifice or transection, which results in complete diaphragmatic paralysis and has been reported in approximately 30% of cases.^4^^,^^5^ Even when the phrenic nerve is preserved, postoperative palsy still occurs in about 5.4% of patients, and preservation efforts also carry the added risk of local recurrence, even with postoperative radiotherapy.^4^ In addition, in some cases, dense peritumoural inflammation prevents safe dissection of the nerve, even in the absence of pathological invasion.^4^

Phrenic nerve palsy can cause atelectasis, pneumonia, sleep-disordered breathing, and pleural effusion, leading to dyspnoea and hypoxia that may require ventilatory support.^1^^,^^5^^,^^6^ Sacrificing the phrenic nerve during thoracic surgery is associated with reduced postoperative respiratory function, including decreased vital capacity, prolonged ventilation, and higher postoperative morbidity and mortality.^6–8^

Several techniques for phrenic nerve reconstruction have been reported, including direct end-to-end anastomosis and interposition nerve grafting. Direct anastomosis can restore diaphragmatic function when the nerve defect is short,^9^ but it is not feasible for long nerve gaps. However, evidence regarding the efficacy of phrenic nerve reconstruction using interposition grafts remains limited, particularly in terms of its impact on diaphragmatic motion and respiratory function.^10^^,^^11^

This study retrospectively evaluated the effect of phrenic nerve reconstruction using intercostal nerve grafts on postoperative diaphragmatic motion and respiratory function, compared with cases without reconstruction.

## METHODS

### Patients and phrenic nerve reconstruction

The study was approved by the institutional review board of Kanagawa Cancer Center (approval number: 2024 Eki-102; date of approval: November 5, 2024). Individual consent for this retrospective analysis was waived. This study was conducted in accordance with the principles of the Declaration of Helsinki and the WMA Declaration of Taipei on ethical considerations regarding health databases. The institutional database used in this study was approved and is continuously monitored by the ethics committee. Eleven consecutive patients underwent combined resection of the phrenic nerve due to thoracic (lung or mediastinal) tumours involving the phrenic nerve between October 2023 and January 2025. Reconstruction was considered in patients who met all of the following conditions:

1. Both proximal and distal phrenic nerve stumps were identifiable and accessible intraoperatively, as successful end-to-end nerve grafting requires clearly defined stumps.
2. Patients with partial or preserved preoperative phrenic nerve function, defined as those showing any diaphragmatic motion on preoperative inspiratory and expiratory chest X-rays, were considered, since complete paralysis leads to total diaphragmatic flaccidity and makes functional recovery after reconstruction unlikely.

**Figure 1.**
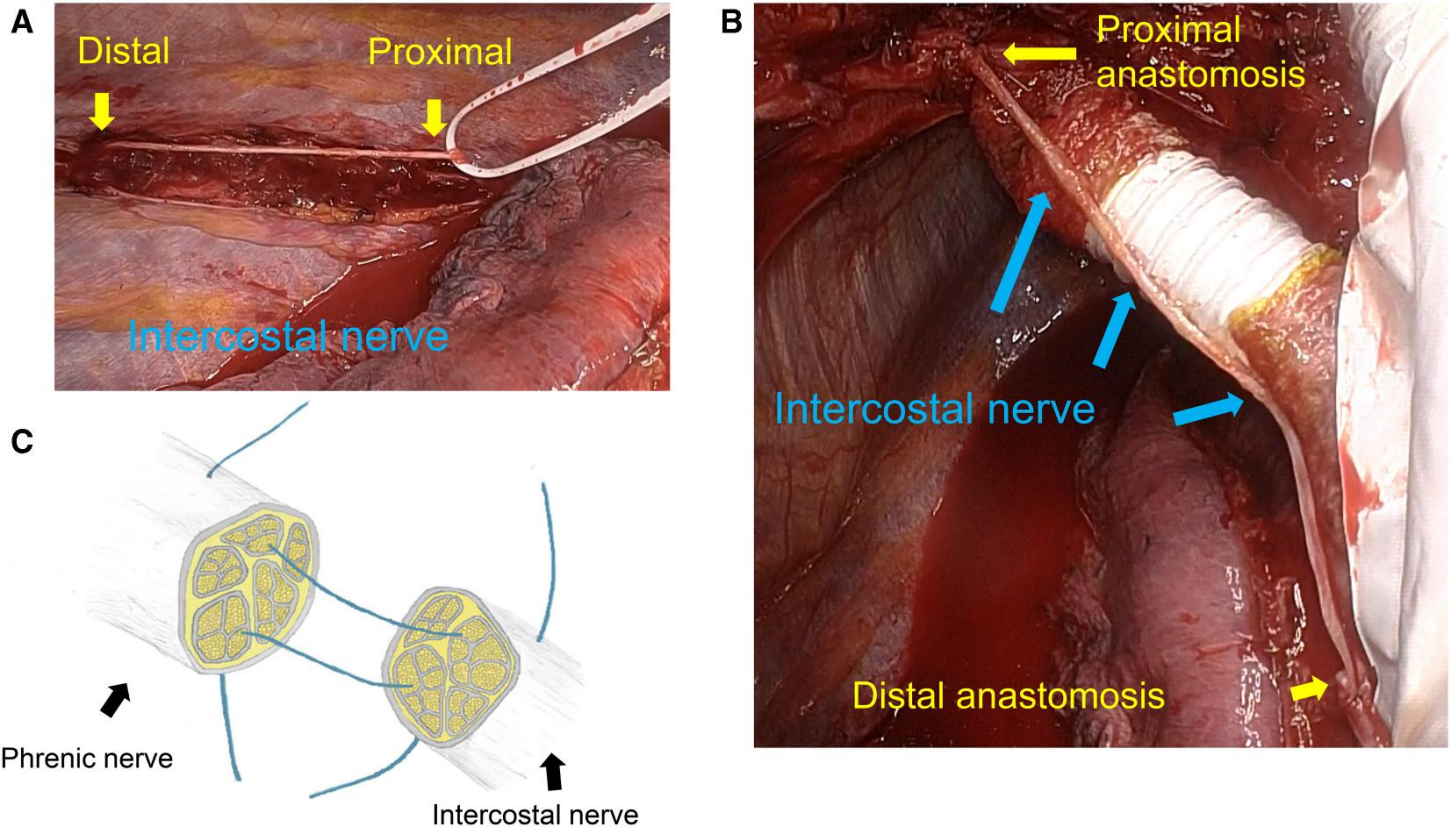
Phrenic Nerve Reconstruction Using an Intercostal Nerve Graft. The intercostal nerve was carefully harvested within the thoracic cavity, cutting the portion nearest the vertebral body as the proximal end and obtaining a length sufficient for the graft (A). The proximal cut end of the phrenic nerve was connected to the proximal end of the intercostal nerve graft, and the distal cut end of the phrenic nerve was connected to the distal end of the intercostal nerve (B). End-to-end anastomosis was performed using 5-0 or 6-0 Prolene sutures (C)

In the reconstruction group, the intercostal nerve was carefully harvested within the thoracic cavity, with the portion closest to the vertebral body considered the proximal end. The nerve was cut at the proximal end, and a length sufficient for the graft was harvested (**Figure 1A**). The proximal stump of the transected phrenic nerve was connected to the proximal end of the intercostal nerve, while the distal stump of the phrenic nerve was connected to the distal end of the intercostal nerve with 5-0 or 6-0 Prolene (Ethicon, Somerville, New Jersey, United States) and secured with 1 or 2 stitches as appropriate (**Figure 1B and C**). At the surgeon’s discretion, the intercostal nerve graft was wrapped with pericardial fat following anastomosis. In the nonreconstruction group, surgery was completed without reconnecting the severed phrenic nerve.

### Evaluation of respiratory function, diaphragmatic movement, and statistical analyses

**Figure 2.**
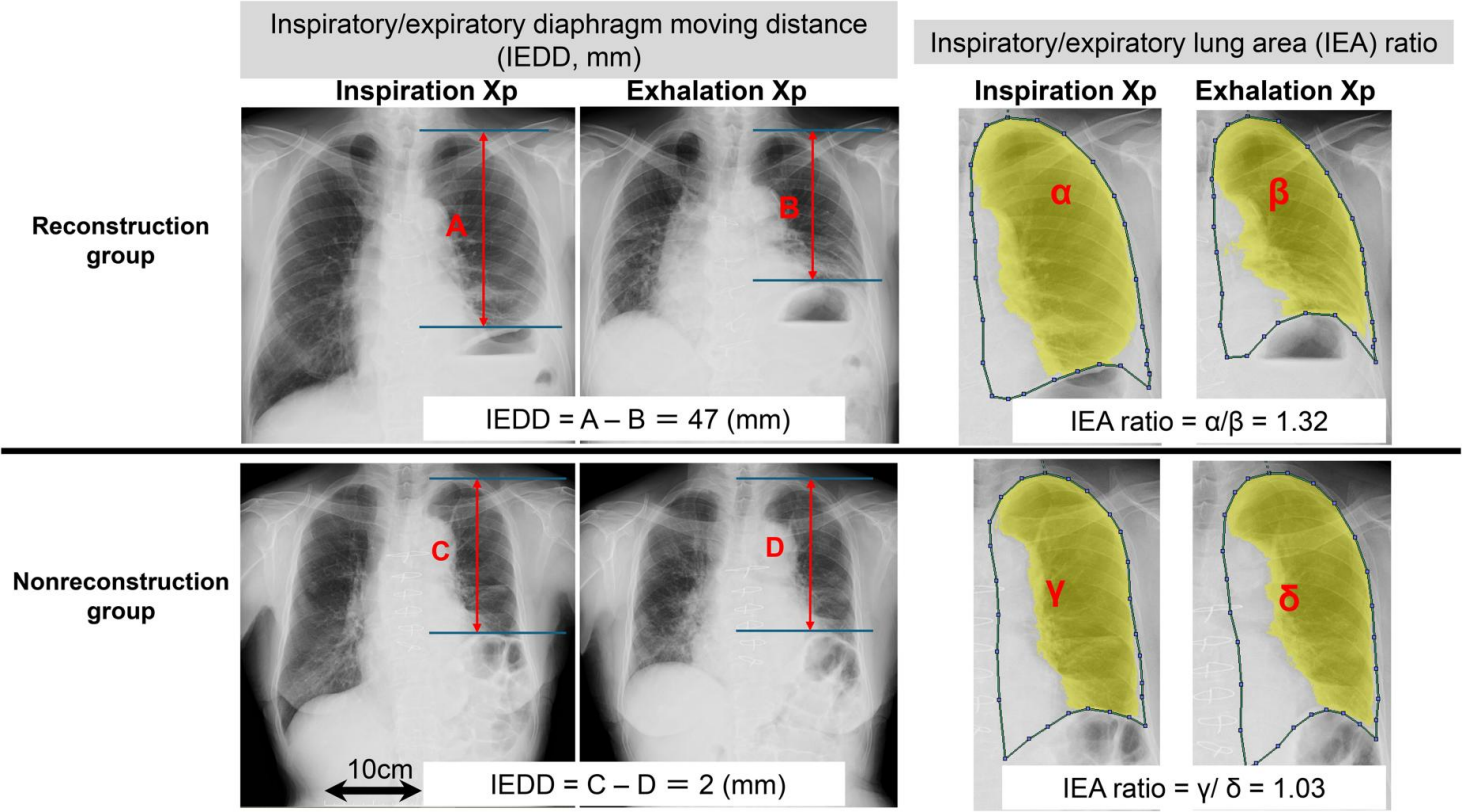
Mesurement of IEDD and IEA ratio on Xp. IEDD and IEA ratio were semiautomatically measured on Xp during the postoperative period using SYNAPSE VINCENT. A-B and α/β show examples of IEDD and IEA ratio measurements in the reconstruction group, respectively. C-D and γ/δ show examples of IEDD and IEA ratio measurements in the nonreconstruction group, respectively. The yellow area represents the lung, and the dotted line indicates the boundary of the thorax

Preoperative respiratory function was measured within 3 months before surgery, and postoperative respiratory function was assessed about 6 months after the operation. Inspiratory and expiratory chest X-rays (Xp) were taken within 1 month, at 1-3 months, and at 4-6 months postoperatively. The inspiratory/expiratory diaphragm movement distance (IEDD) and the inspiratory/expiratory lung area (IEA) ratio were semiautomatically calculated on Xp at each postoperative time point (within 1 month, 1-3 months, and 4-6 months) using SYNAPSE VINCENT (Fujifilm Medical Co., Ltd, Tokyo, Japan) by T.I. (**Figure 2**). The primary aim of this study was to compare postoperative IEDD, IEA ratio, percentage predicted vital capacity (%VC), and percentage predicted forced expiratory volume in 1 s (%FEV1.0) between the reconstruction and nonreconstruction groups. In the present study, a diaphragmatic movement of <10 mm was used for diagnosing diaphragmatic dysfunction.^12^ Continuous variables and categorical variables were compared between the groups using the Mann-Whitney *U* test or Welch test and Fisher’s exact test, respectively. Statistical significance was defined as *P *< .05. All statistical analyses were conducted using EZR on R Commander (version 1.30; Saitama Medical Center, Jichi Medical University, Saitama, Japan), a graphical user interface for R (The R Foundation for Statistical Computing, Vienna, Austria).

## RESULTS

**Figure 3.**
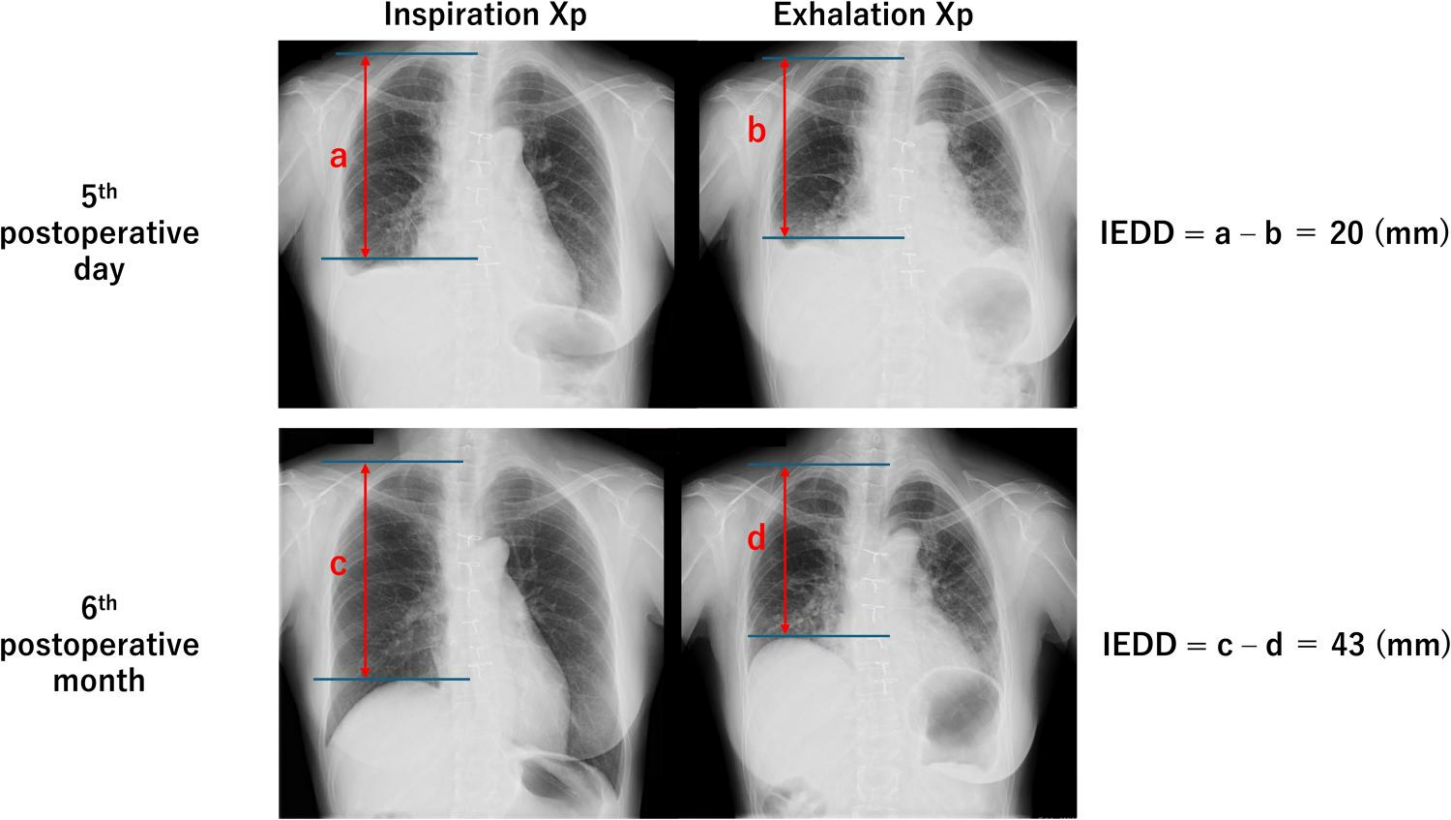
Diaphragmatic Movement on Xp After Phrenic Nerve Reconstruction. The earliest postoperative improvement in diaphragmatic movement (Over 10 mm on Xp) was observed in a patient who had undergone right phrenic nerve reconstruction, with an IEDD of 20 mm on day 5 and 43 mm at 6 months

Among the 11 patients with resected phrenic nerves, 8 (72.7%) were assigned to the reconstruction group and 3 (27.3%) to the nonreconstruction group. In the reconstruction group, the median length of the resected phrenic nerve was 8 cm (interquartile range, 8-11 cm) and the reconstruction time was 19 min (interquartile range, 17-21 min). There were no significant differences between the groups in age (75 vs 81 years, *P *= .357), sex (male, 5 vs 2 patients, *P *= 1.000), or type of the tumour (mediastinum tumour, 5 vs 2 patients, *P *= 1.000) (**Table 1**). Diaphragm elevation was noted on preoperative Xp in 4 patients in the reconstruction group and 1 patient in the nonreconstruction group (*P *= 1.000). Diaphragmatic motion of ≥10 mm on postoperative Xp was observed as early as 5 days after surgery in the reconstruction group (**Figure 3**). No postoperative complications were observed following intercostal nerve harvesting (**Table 1**).

**Table 1.** Comparison of Clinicopathological Characteristics Between the Reconstruction and Nonreconstruction Groups

Variable	Reconstruction (n = 8)	Nonreconstruction (n = 3)	*P*-values^a^
Age (years), median (IQR),	75 (55-79)	81 (73-83)	.357^b^
Male, *n* (%),	5 (62.5)	2 (66.7)	1.000
Smoking history (ever), *n* (%)	3 (37.5)	1 (33.3)	1.000
Chronic lung disease history, *n* (%)	1 (12.5)	1 (33.3)	.491
Type of the thoracic tumour, *n* (%)			
Mediastinum tumour	5 (62.5)	2 (66.7)	
Lung cancer	3 (37.5)	1 (33.3)	1.000
Tumour size (cm), mean (SD)	6.6 (2.7)	6.4(2.1)	.896^c^
Diaphragm elevation on preoperative Xp,^d^ *n* (%)	4 (50)	1 (33.3)	1.000
Phrenic nerve transection, left side, *n* (%)	6 (75.0)	2 (66.7)	1.000
Length of the resected phrenic nerve (cm), median (IQR)	8 (8-11)		
Reconstruction time (min), median (IQR)	19 (17-21)		
Intercostal nerve graft covered with pericardial fat tissue, *n* (%)	3 (37.5)		
Lung resection, *n* (%)	8 (100)	2 (66.7)	.273
Complicated pericardium resection, *n* (%)	7 (87.5)	2 (66.7)	.491
Postoperative complication,^e^ *n* (%)			
Pleural effusion grade IIIa	2 (25.0)	1 (33.3)	1.000
Atrial fibrillation grade II	0	1 (33.3)	.273
Pneumonia grade II	2 (25.0)	0	1.000
Chest tube removal (postoperative day), mean (SD)	3.5 (1.4)	4.3 (1.5)	.465^c^
Discharge (postoperative day), median (IQR)	9 (8-12)	7 (7-13)	.757^b^
R1 resection, *n* (%)	0	1 (33.3)	.273
Pathology, *n* (%)			
Thymic cancer	1 (12.5)	1 (33.3)	
Thymoma	2 (25.0)	0	
Lung cancer	3 (37.5)	1 (33.3)	
Other cancer	2 (25.0)	1 (33.3)	1.000

a Fisher’s exact test.

b Mann-Whitney *U* test.

c Welch test.

d Right side, ≥4 cm; left side, elevation greater than the right side.

e Clavien-Dindo classification.

Abbreviations: IQR = interquartile range; Xp = chest X-ray.

Diaphragmatic motion of ≥10 mm was seen in 4 patients (50%) within 1 month and in 7 patients (87.5%) at 4-6 months postoperatively in the reconstruction group. The mean IEDD on the transected side for the reconstruction and nonreconstruction groups was 19.8 vs 4.1 mm (*P *= .013) at 1-3 months and 19.8 vs 4.4 mm (*P *= .031) at 4-6 months, respectively. The mean IEA ratio for the 2 groups was 1.16 vs 1.04 (*P *= .026) at 1-3 months and 1.19 vs 1.05 (*P *= 0.031) at 4-6 months postoperatively. The IEDD and IEA ratio on the intact side showed no significant differences between the groups at any postoperative time point (**Table 2**).

**Table 2.** Comparison of Postoperative Inspiratory/Expiratory Diaphragm Movement Distance (IEDD) and Inspiratory/Expiratory Area (IEA) Ratio between the Reconstruction and Nonreconstruction Groups

Variable			Reconstruction (n = 8)	Nonreconstruction (n = 3)	*P*-values^a^
Diaphragmatic movement of ≥10 mm, *n* (%)		Within 1 m	4 (50%)	0	
		1-3 m	6 (75%)	0	
		4-6 m	7 (87.5%)	1 (33.3%)	
IEDD (mm), mean (SD)	Transected side	1-3 m	19.8 (13.4)	4.1 (2.6)	.013
		4-6 m	19.8 (15.9)	4.4 (3.9)	.031
	Untransected side	1-3 m	43.3 (17.7)	31.6 (5.7)	.133
		4-6 m	40.3 (24.1)	26.9 (12.2)	.262
IEA ratio, mean (SD)	Transected side	1-3 m	1.16 (0.11)	1.04 (0.04)	.026
		4-6 m	1.19 (0.15)	1.05 (0.03)	.031
	Untransected side	1-3 m	1.41 (0.24)	1.25 (0.11)	.164
		4-6 m	1.42 (0.27)	1.29 (0.10)	.283

a Welch test.

Abbreviations: IEA = inspiratory/expiratory area; IEDD = inspiratory/expiratory diaphragm movement distance; m = month.

No significant differences in preoperative respiratory function were observed between the 2 groups (**Table 3**). Similarly, the timing of postoperative respiratory function testing did not differ significantly (4 vs 6 months after surgery, *P *= .442). The reconstruction group showed higher %VC (78% vs 56%, *P *= .008) and %FEV1 (72% vs 45%, *P *< .001) than the nonreconstruction group (**Table 3**).

**Table 3.** Comparison of Preoperative and Postoperative Respiratory Function Between the Reconstruction and Nonreconstruction Groups

Variable		Reconstruction (n = 8)	Nonreconstruction (n = 3)	*P-*values^a^
Preoperative respiratory function	VC (mL), mean (SD)	2850 (623)	2240 (1100)	.410
	%VC (%), mean (SD)	97 (24)	78 (20)	.231
	FVC (mL), mean (SD)	2855 (610)	2077 (977)	.300
	%FVC (%), mean (SD)	91.7 (20)	67.4 (20)	.158
	FEV1 (mL), mean (SD)	2121 (448)	1603 (723)	.342
	%FEV1 (%), mean (SD)	88 (22)	68 (20)	.255
	FEV1% (%), mean (SD)	75 (11)	78 (4)	.556
Postoperative respiratory function	VC (mL), mean (SD)	2316 (545)	1550 (490)	.088
	%VC (%), mean (SD)	78 (16)	56 (6)	.008
	FVC (mL), mean (SD)	2331 (537)	1480 (494)	.069
	%FVC (%), mean (SD)	75 (15)	50 (6)	.003
	FEV1 (mL), mean (SD)	1755 (331)	1033 (319)	.033
	%FEV1 (%), mean (SD)	72 (14)	45 (3)	<.001
	FEV1% (%), mean (SD)	76 (8)	70 (3)	.106
Period of postoperative respiratory function test, months (SD)		4 (2)	6 (2)	0.442

a Welch test.

Abbreviations: FEV1 = forced expiratory volume in 1 s; FEV1% = FEV1/FVC ratio; %FEV1 = percentage predicted forced expiratory volume in 1 s; FVC = forced vital capacity; %FVC = percentage predicted forced vital capacity; VC = vital capacity; %VC = percentage predicted vital capacity.

## DISCUSSION

In this study, phrenic nerve reconstruction using an intercostal nerve graft was successfully performed in patients requiring extensive nerve resection, with a median defect length of 8 cm. The procedure was feasible and safe, taking approximately 19 min, and was completed without complications related to intercostal nerve harvesting. Early diaphragmatic motion was observed as early as postoperative day 5, and 87.5% of patients demonstrated recovery within 6 months. Postoperative respiratory function, including %VC and %FEV1, was significantly better in the reconstruction group, suggesting that intercostal nerve grafting effectively mitigates diaphragmatic dysfunction and helps preserve pulmonary capacity.

The intercostal nerves are motor nerves that supply various muscles and contribute to respiration and posture, including trunk flexion, extension, and rotation.^11^ Similar to the phrenic nerve, intercostal nerves release acetylcholine as a neurotransmitter to stimulate muscle contraction, making them physiologically compatible.^13^ Intercostal nerve grafting for phrenic nerve reconstruction has also been applied in patients with cervical spinal injuries to restore diaphragmatic motion and reduce ventilator dependence.^5^^,^^13–16^ Other nerve grafts, including those of the sural nerve and trapezius branch of the spinal accessory nerve, have been used for phrenic nerve reconstruction.^5^^,^^17^^,^^18^ However, the intercostal nerve is considered the most suitable because it is easily identified and safely harvested from the same surgical field. It also closely matches the phrenic nerve in diameter and causes minimal donor-site morbidity.^11^

Only a limited number of cases have reported phrenic nerve reconstruction using an intercostal nerve graft after phrenic nerve resection in patients with intrathoracic tumours. Kawashima et al described diaphragmatic motion on inspiratory and expiratory Xp at 2-27 days postoperatively, and Tamagawa et al reported similar findings at 3 months.^10^^,^^11^ In our study, patients who underwent phrenic nerve reconstruction began to show diaphragmatic movement as early as postoperative day 5, with gradual improvement over the first 6 months. Although not routinely applied, postoperative respiratory rehabilitation may further promote early recovery.^19^

The diaphragm accounts for approximately 80% of the muscular power of respiration.^20^ Unilateral diaphragmatic paralysis reduces predicted VC, FEV1, and FVC by 25%-40%, 30%, and 30%-50%, respectively.^20–23^ In our cohort, these functions were reduced by only 19%, 17%, and 18% at 4-6 months postoperatively. In the nonreconstruction group, %VC, %FEV1, and %FVC were 56%, 45%, and 50%, compared with 78%, 72%, and 75% in the reconstruction group (**Table 3**). These results suggest that phrenic nerve reconstruction preserves postoperative respiratory function, particularly in patients with pre-existing pulmonary compromise.

Most patients showed diaphragmatic motion by 6 months, with potential for further improvement over longer follow-up. Summerhill et al^23^ suggested that diaphragmatic function gradually improves over the long term in patients with diaphragm weakness. This functional recovery is likely driven by coordinated processes of phrenic nerve regeneration after grafting: Schwann cells in the graft, and those in the proximal and distal nerve stumps, dedifferentiate, proliferate, and secrete neurotrophic factors that support axonal growth and remyelination.^24^^,^^25^ Both the transected phrenic nerve stump and autologous nerve graft supply endoneurial tubes and a supportive extracellular matrix, guiding the slowly regenerating axons across the anastomosis and gradually restoring nerve conduction and diaphragmatic motion.^26^^,^^27^

Various techniques for phrenic nerve anastomosis have been reported. Krieger et al used the fourth intercostal nerve with 7-0 Prolene and interrupted epineural sutures,^13^ while Frasca et al anastomosed using 8-0 Prolene.^28^ Tamagawa et al performed intercostal-to-phrenic nerve anastomosis with a figure-of-8 suture using 6-0 thread,^10^ and Kawashima et al used 4-0 or 6-0 PDS II with Macro Needle Holders.^11^ Schoeller et al reported reconstruction with a sural nerve graft using 8-0 Ethilon microsurgical sutures.^17^ A sufficiently long graft is critical to ensure tension-free anastomosis and preserve nerve function.^13^

In this study, the intercostal nerve graft was covered with pericardial fat tissue in 3 patients. Yoshitani et al reported experimental findings from diaphragmatic nerve reconstruction in dogs, demonstrating that placing the phrenic nerve within a pericardial fat pad improved diaphragmatic function compared to cases without the fat pad.^29^ Kaufman et al routinely used pericardial fat pads to wrap interposition nerve grafts.^5^ Krieger et al retained a 5-mm cuff of tissue around the intercostal nerve to help maintain its blood supply during anastomosis.^13^ Since pericardial fat tissue is believed to promote neovascularization and release cytokines that aid tissue repair,^5^ wrapping nerves may enhance diaphragmatic function after phrenic nerve reconstruction.

Diaphragm plication is another approach for treating diaphragmatic paralysis, placing the diaphragm in an inspiratory position to improve lung function and relieve dyspnoea.^6^^,^^30^^,^^31^ However, it carries risks of postoperative complications, including pneumonia, atrial fibrillation, and, rarely, mortality.^30^^,^^32^ Its benefits in patients undergoing phrenic nerve resection during thoracic tumour surgery remain controversial. Intraoperative prophylactic diaphragmatic plication may help preserve lung function and reduce the risk of postoperative ventilator dependence in patients undergoing phrenic nerve transection,^33^^,^^34^ but its routine application requires further evaluation.

This study has several limitations. First, its retrospective design and small sample size may have introduced selection bias. The decision to perform phrenic nerve reconstruction was made intraoperatively, as the procedure requires the identification of both proximal and distal nerve stumps; this decision could have been influenced by factors such as tumour size, preoperative respiratory function, extent of phrenic nerve paralysis, and anatomical accessibility, potentially confounding the outcomes. Second, the postoperative follow-up was relatively short (approximately 6 months), and given that nerve regeneration and functional recovery may continue over several years,^35^ longer-term evaluation is necessary. Third, postoperative rehabilitation protocols were not standardized, and quality-of-life outcomes were not assessed. Future multicentre prospective studies with larger cohorts and extended follow-up are warranted to validate the long-term efficacy of phrenic nerve reconstruction.

## CONCLUSION

This study showed that phrenic nerve reconstruction using intercostal nerve grafts is a feasible and safe procedure that mitigates postoperative diaphragmatic dysfunction and helps preserve respiratory function. These findings highlight the potential clinical benefit of intercostal nerve grafts for patients requiring phrenic nerve resection during thoracic tumour surgery. Further studies are warranted to evaluate long-term functional outcomes.

## Data Availability

The data underlying this article will be shared on reasonable request to the corresponding author.
